# Changes in Gene Expression Profile with Age in SAMP8: Identifying Transcripts Involved in Cognitive Decline and Sporadic Alzheimer’s Disease

**DOI:** 10.3390/genes15111411

**Published:** 2024-10-31

**Authors:** Christian Griñán-Ferré, Iris Valeria Servin-Muñoz, Verónica Palomera-Ávalos, Carmen Martínez-Fernández, Júlia Companys-Alemany, Amalia Muñoz-Villanova, Daniel Ortuño-Sahagún, Mercè Pallàs, Aina Bellver-Sanchis

**Affiliations:** 1 Department of Pharmacology and Therapeutic Chemistry, Institut de Neurociències-Universitat de Barcelona, Av. Joan XXIII 27-31, 08028 Barcelona, Spain; christian.grinan@ub.edu (C.G.-F.); veropalomera@ub.edu (V.P.-Á.); cmartinezf14@gmail.com (C.M.-F.); juliacompanysalemany@gmail.com (J.C.-A.); amalia-mv@hotmail.com (A.M.-V.); pallas@ub.edu (M.P.); 2 Centro de Investigación en Red, Enfermedades Neurodegenerativas (CIBERNED), Instituto de Salud Carlos III, 28029 Madrid, Spain; 3 Laboratorio de Neuroinmunología Molecular, Instituto de Investigación de Ciencias Biomédicas (IICB) CUCS, Universidad de Guadalajara, Jalisco 44340, Mexico; iris.servin@alumnos.udg.mx (I.V.S.-M.); daniel.ortuno@academicos.udg.mx (D.O.-S.)

**Keywords:** aging, cognitive decline, Alzheimer’s disease, SAMP8, transcriptomics

## Abstract

**Background:** The senescence-accelerated mouse 8 (SAMP8) represents a model for Alzheimer’s disease (AD) research because it exhibits age-related learning and memory impairments consistent with early onset and rapid progression of senescence. To identify transcriptional changes during AD progression, in this study, we analyzed and compared the gene expression profiles involved in molecular pathways of neurodegeneration and cognitive impairment in senescence-accelerated resistant 1 (SAMR1) and SAMP8 mice. **Methods:** In total, 48 female SAMR1 and SAMP8 mice were randomly divided into six groups (SAMR1 and SAMP8 at 3, 7, and 9 months of age). Microarray analysis of 22,000 genes was performed, followed by functional analysis using Gene Ontology (NCBI) and examination of altered molecular pathways using the KEGG (Kyoto Encyclopedia of Genes and Genomes). **Results:** SAMP8 mice had 2516 dysregulated transcripts at 3 months, 2549 transcripts at 7 months, and 2453 genes at 9 months compared to SAMR1 mice of the same age. These accounted for 11.3% of the total number. This showed that with age, the gene expression of downregulated transcripts increases, and that of over-expressed transcripts decreases. Most of these genes were involved in neurodegenerative metabolic pathways associated with Alzheimer’s disease: apoptosis, inflammatory response, oxidative stress, and mitochondria. The qPCR results indicated that *Ndufs4*, *TST/Rhodanese*, *Wnt3*, and *Sema6a* expression was differentially expressed during aging. **Conclusions:** These results further revealed significant differences in gene expression profiles at different ages between SAMR1 and SAMP8 and showed alteration in genes involved in age-related cognitive decline and mitochondrial processes, demonstrating the relevance of the SAMP8 model as a model for sporadic AD.

## 1. Introduction

As the life expectancy of the world’s population has increased, so has the incidence of cognitive impairment and dementia, representing a public health problem. Aging of the brain itself is the leading risk factor for cognitive decline and neurodegenerative diseases such as Alzheimer’s disease (AD), a progressive neurodegenerative disorder associated with cognitive decline, memory impairment, and brain atrophy. Its physiopathology consists mainly of aggregates of the peptide Aβ and hyperphosphorylated tau, leading to deposits of Aβ peptides and intracellular neurofibrillary tangles [1,2]. There are two types of AD: familial AD (FAD), which accounts for only 5% of cases of AD [3], and sporadic AD (SAD), which represents 95% of Alzheimer’s cases. The cause of FAD is mutations in *PSEN1* and *PSEN2* genes, but SAD is a multifactorial pathology; some of the risk factors are allelic variations of apolipoprotein E (ApoE), metal exposure, traumatic brain injury (TBI), cardiovascular disease, depression, stress, sleep disorders, and age, which is one of the main risk factors [4,5,6].

Recent studies have shed further light on the complex interplay between aging and neurodegeneration in AD. For instance, Griñán-Ferré et al. (2018) [7] demonstrated that epigenetic alterations, particularly changes in DNA methylation and histone modifications, are crucial in accelerated aging with short life expectancy (9.6 months) and cognitive decline appearance as early as 3 months of age in SAMP8. These epigenetic changes have been found to affect genes involved in neuroplasticity, oxidative stress response, and inflammatory processes, all of which have been linked to AD development. Furthermore, Akiguchi et al. (2017) [8] highlighted the importance of cerebrovascular alterations in the SAMP8 model and showed that these mice exhibit early-onset and progressive cerebral hypoperfusion, blood–brain barrier (BBB) disruption, and white matter lesions, reflecting the vascular component of human AD. This finding emphasizes the value of the SAMP8 model for studying the vascular contributions to cognitive impairment and dementia. In addition, a study by Cheng et al. (2019) [9] revealed significant alterations in the gut microbiome of SAMP8 mice, suggesting a possible link between gut dysbiosis and neurodegeneration. This fits with the growing body of evidence supporting the gut–brain axis in AD pathogenesis and offers new opportunities for therapeutic interventions. The senescence-accelerated mouse (SAM) has been used primarily for this purpose over the past decade. This is a mouse model for accelerated aging developed by Dr. Takeda in 1981 by phenotypic selection from the AKR/J strain [10]. The substrains SAMP (senescence-accelerated prone) and SAMR (senescence-accelerated resistant) were developed from the SAM strain [11]. The SAMP8 substrain is an ideal strain for the study of early age-related diseases as it closely resembles the age-related changes found in AD patients, such as cognitive impairment, learning and memory deficits, β-amyloid (Aβ) peptide aggregates, hyperphosphorylated tau, gliosis in astrocytes, alterations in the cholinergic system, and epigenetic modifications. All the above enables the study of SAD since they are not caused by mutations in the *PSEN1* and *PSEN2* genes (*encoding presenilin 1 and 2* (*PS1 and PS2*)) as is the case in the models used to study FAD [12]. In contrast, the SAMR1 substrain represents the standard aging control. However, as these strains are phenotypically selected, the cause of the accelerated aging process in SAMP8 mice are largely unknown.

Therefore, uncovering the molecular links between age-related cognitive decline and neurodegeneration becomes essential for preventing age-related diseases. Consequently, we performed a transcriptomic analysis and described the differential genetic landscape between SAMR1 and SAMP8 mice at different ages with the aim of finding candidate genes that may be involved in the accelerated and pathological aging process in SAMP8. All of this has led us to finally characterize the expression profile of the genes that change with age in this well-established mouse model of AD.

## 2. Materials and Methods

### 2.1. Animals

Female SAMR1 (n = 24) and SAMP8 (n = 24) mice aged 3, 7, and 9 months, were used for the experiments. Animals were given free access to food and water and maintained under standard temperature conditions (22 ± 2 °C) and 12:12 h light:darkness (300 lx/0 lx). The studies were conducted according to the institutional guidelines for the care and use of laboratory animals established by the University of Barcelona’s Animal Experimentation Ethics Committee (CEEA).

### 2.2. Brain Processing and RNA Extraction

The animals were euthanized via cervical dislocation. Brains were immediately removed from the skull, and the hippocampus was isolated, frozen in powdered dry ice, and kept at −80 °C until RNA isolation. Whole RNA isolation was performed using TRIzol reagent according to the manufacturer’s instructions. RNA yield, purity, and quality were determined using a NanoDrop™ ND-1000 (Thermo Scientific, Waltham, MA, USA) and an Agilent 2100B Bioanalyser (Agilent Technologies, Singapore). RNA with a 260/280 ratio of more than 1.9 was selected. Reverse transcription-polymerase chain reaction (RT-PCR) was performed in accordance with the following procedure: 2 μg of messenger RNA (mRNA) was reverse transcribed using the High-Capacity DNA Reverse Transcription Kit (Applied Biosystems, Waltham, MA, USA).

### 2.3. Analysis Microarray

Complementary DNA was synthesized using 10 ug of extracted total RNA and double-labeled with the fluorescent markers dUTP-Cy3 and dUTP-Cy5. The cDNA was hybridized to a chip containing 22,000 oligo mouse array, as previously described [13,14]. Quantification of array images was performed on the ScanArray 4000, and mean Cy3 and Cy5 density values and mean Cy3 and Cy5 background values were calculated using ArrayPro Analyzer 6.3, released in 2011 software (Media Cibernetics, Singapore). Microarray data were analyzed using the GenArise software v2.0 developed by the Computational Unit of the Institute of Cell Physiology at UNAM [15]. This software identifies differentially expressed genes by calculating the Z-score value, which indicates the number of standard deviations by which each gene deviates from its mean value.

### 2.4. Gene Ontology and KEGG Pathway Enrichment Analysis

Gene ontology for cellular components, biological processes, and molecular function, as well as KEGG pathway enrichment analyses, were extracted from the Database for Annotation Visualization and Integrated Discovery (DAVID) [16] for the selected up- and downregulated genes with a Z-Score > ±1.5.

### 2.5. Network of Protein–Protein Interaction and Functional Analysis

We used the Search Tool for the Retrieval of Interacting Genes (STRING) database to analyze the interactions between the genes with altered expression.

### 2.6. Validation of the Microarray by Quantitative Real-Time PCR

Quantitative real-time PCR (qPCR) was used to validate selected genes from the microarray data results. SYBR Green real-time PCR was conducted on a Step OnePlus detection system (Applied-Biosystems) using the SYBRGreen PCR Master Mix (Applied-Biosystems). Each reaction mix contained 7.5 μL of complementary DNA (cDNA) (concentration of 2 μg), 0.75 μL of each primer (forward and reverse) (concentration of 100 nM), and 7.5 μL of SYBRR Green PCR Master Mix (2X). Data were analyzed by the comparative cycle threshold (Ct) method (ΔΔCt), with housekeeping genes employed to adjust for differences in loading and sample preparation. The expression levels were normalized using β-actin as the reference gene. Each sample (n = 4 per group) was analyzed in duplicate, and the results represent the n-fold difference in transcript levels between the different groups.

### 2.7. Statistical Analysis

Data analysis was performed using GraphPad Prism ver. 9 statistical software. Data are presented as mean ± standard error of the mean (SEM) of 4 samples per group. All data were tested for normal distribution and equal variance. Means were compared using a one-way analysis of variance (ANOVA) followed by a Tukey post hoc test. The comparison between the groups was also performed with a two-tailed Student’s *t*-test for independent samples if required. Statistical significance was assumed if the *p* values were <0.05. Statistical outliers were identified using the Grubs test and removed from the analysis where appropriate.

## 3. Results

### 3.1. Changes in the Gene Expression Profiles of SAMR1 and SAMP8 Mice at Different Ages

**Figure 1:**
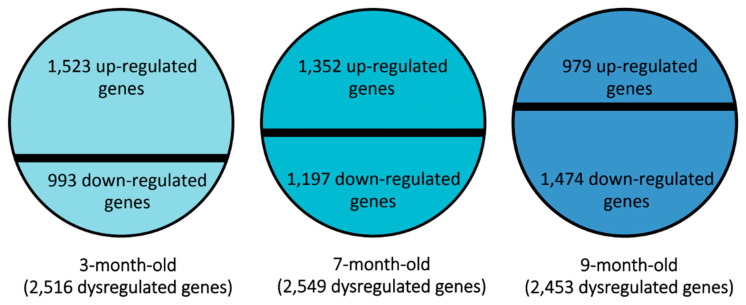
Number of genes differentially expressed in the hippocampus of SAMP8 mice compared to SAMR1 mice at 3, 7, and 9 months of age.

We obtained a list of significantly upregulated and downregulated genes from the microarray findings. Comparisons between the two strains used in this study showed that several genes were differentially expressed. Specifically, we found that at 3 months of age, a total of 2516 genes were significantly dysregulated in SAMP8 mice, with 1523 genes being upregulated and 993 downregulated compared to SAMR1 mice. Interestingly, at 7 months of age, 2549 genes were significantly dysregulated in SAMP8 mice, 1352 were upregulated, and 1197 were downregulated. At 9 months of age, 2453 genes were significantly altered, of which 979 were upregulated and 1474 downregulated compared to SAMR1 mice based on Z-Score criteria (Table 1, Figure 1 and Supplementary Table S1). Interestingly, at 3 months of age, the number of upregulated genes is higher than the number of downregulated genes; at 7 months of age, this number is balanced; and at 9 months of age, the number is reversed; it means that the number of upregulated genes is lower than the number of downregulated genes. These data suggest that at early stages, an attempt is made to compensate for the pathological state by trying to achieve homeostasis. However, with increasing age, a state is reached in which there is a loss of function of the proteins encoded by various genes with altered expression. We next focused on cellular components, biological processes, and molecular functions associated with these altered expression genes to select genes of interest.

### 3.2. Gene Ontology (GO) Enrichment Analysis

**Figure 2:**
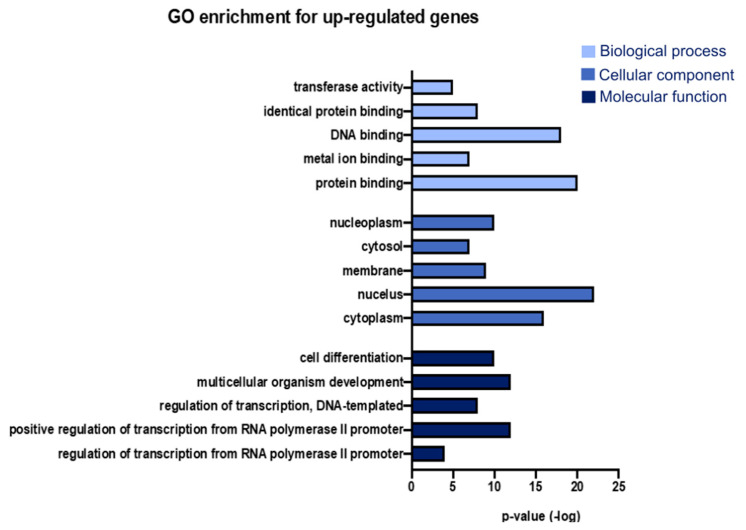
Terms from the GO analysis that summarize the highest number of upregulated genes. Fifteen terms from the GO analysis were collected and integrated into a bar chart; the *Y*-axis represents the *p*-value of the statistic. GO, Gene Ontology.

**Figure 3:**
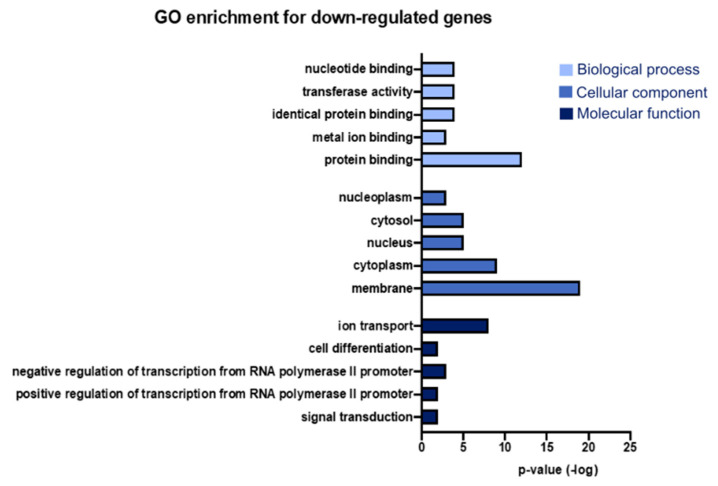
Terms from the GO analysis with the highest number of downregulated genes. Fifteen terms from the GO analysis were collected and integrated into a bar chart; the *Y*-axis represents the *p*-value of the statistic. GO, Gene Ontology.

We performed GO enrichment analysis using the DAVID database to identify the cellular components and biological and molecular functions of the differentially expressed genes in the SAMP8 model [16]. All genes with a Z-score greater than 1.5 or less than −1.5 at the three ages (3, 7, and 9 months) were selected for this analysis. We identified functional and cellular component categories of dysregulated genes. The upregulated genes were highly associated with cytoplasm, nucleus, membrane (identified in cellular component analysis), regulation of transcription by RNA polymerase II promoter and positive regulation of transcription by RNA polymerase II promoter, regulation of transcription, DNA-templated (identified in biological pathway analysis), protein binding, metal ion binding, and DNA binding (identified in molecular function analysis) (Figure 2 and Supplementary Table S2). On the other hand, the downregulated genes were more associated with membrane, cytoplasm, nucleus (identified during cellular component analysis), signal transduction, positive regulation of transcription by RNA polymerase II promoter, negative regulation of transcription by RNA polymerase II promoter (identified during biological pathway analysis), protein binding, metal ion binding, and identical protein binding (identified during molecular function analysis) (Figure 3). In the analysis, we can observe that the functional categories of transferase activity, identical protein binding, metal binding, protein binding, cell differentiation, and positive transcriptional regulation by the RNA polymerase II promoter were highly represented in both groups, indicating a significant change in these functional categories in both directions in the SAMP8 mouse model.

### 3.3. KEGG Pathway Analysis

**Figure 4:**
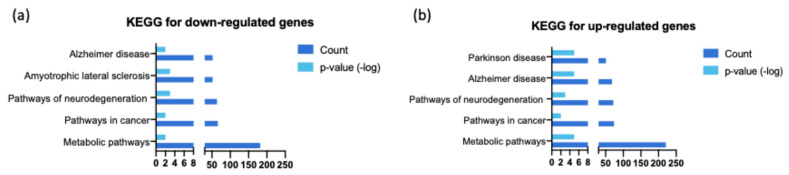
KEGG pathway analysis of upregulated genes in SAMP8 mice. (**a**) Pathways with genes that were upregulated. (**b**) Pathways involving genes that were downregulated in the SAMP8 mouse. We see that the pathways with these genes, both upregulated and downregulated, can be summarized as pathways for neurodegenerative disease, pathways for cancer, and metabolic pathways.

The KEGG database was used to determine enriched pathways of differentially expressed genes in the SAMP8 mouse (Figure 4). Differentially expressed genes obtained at the three ages analyzed (3, 7, and 9 months) with a Z-score greater than 1.5 or less than −1.5 were selected for this analysis. In this analysis, we found enriched signaling pathways mainly associated with three categories: metabolic pathways, neurodegenerative diseases, and cancer pathways, which correspond to the most upregulated and downregulated differentially expressed genes. Metabolic pathways were the most altered category, suggesting widespread changes in cellular metabolism in SAMP8 mice. Altered metabolic pathways likely contribute to the accelerated aging phenotype and increased susceptibility to neurodegenerative processes. When analyzing the over- and under-expressed genes in relation to neurodegenerative disease pathways, AD pathways were particularly strongly represented, underlining the importance of the SAMP8 model for Alzheimer’s research. In addition, other neurodegenerative diseases, such as Parkinson’s disease or amyotrophic lateral sclerosis, were also strongly represented, indicating common mechanisms in the process of neurodegeneration. Genes involved in these signaling pathways may contribute to the cognitive impairments and neuropathological features observed in SAMP8 mice. Finally, the involvement of cancer-related signaling pathways was an interesting finding, highlighting possible links between aging, neurodegeneration, and cancer-associated processes. This could reflect dysregulation of cell cycle control, apoptosis, or DNA repair mechanisms that occur in cancer and neurodegenerative processes (Figure 4).

### 3.4. Protein–Protein Interaction (PPI) Network Integration

**Figure 5:**
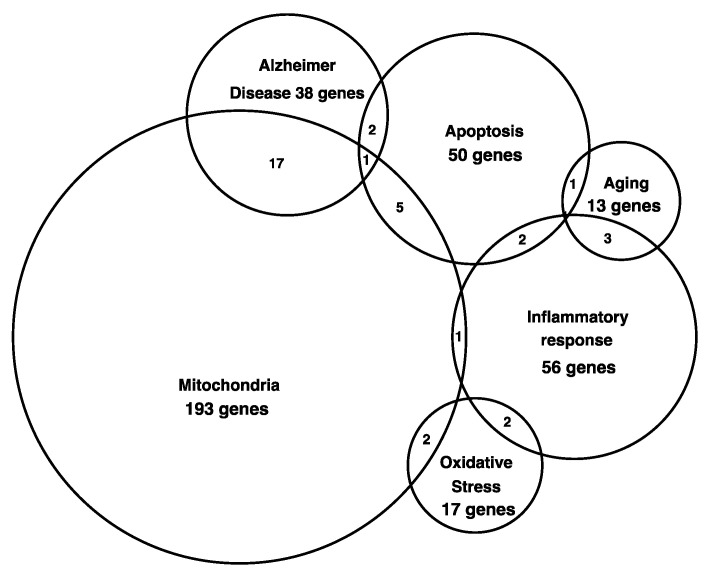
Venn diagram of upregulated and downregulated genes during 3, 7, and 9 months. This diagram shows the relationship between genes involved in more than one function. The function where most genes were altered in the hippocampus of SAMP8 mice were genes with mitochondrial function, which have 17 genes related to Alzheimer’s disease.

Interestingly, some genes were upregulated or downregulated at more than one age, indicating their role in the aging process (Table 2 and Figure 5).

**Figure 6:**
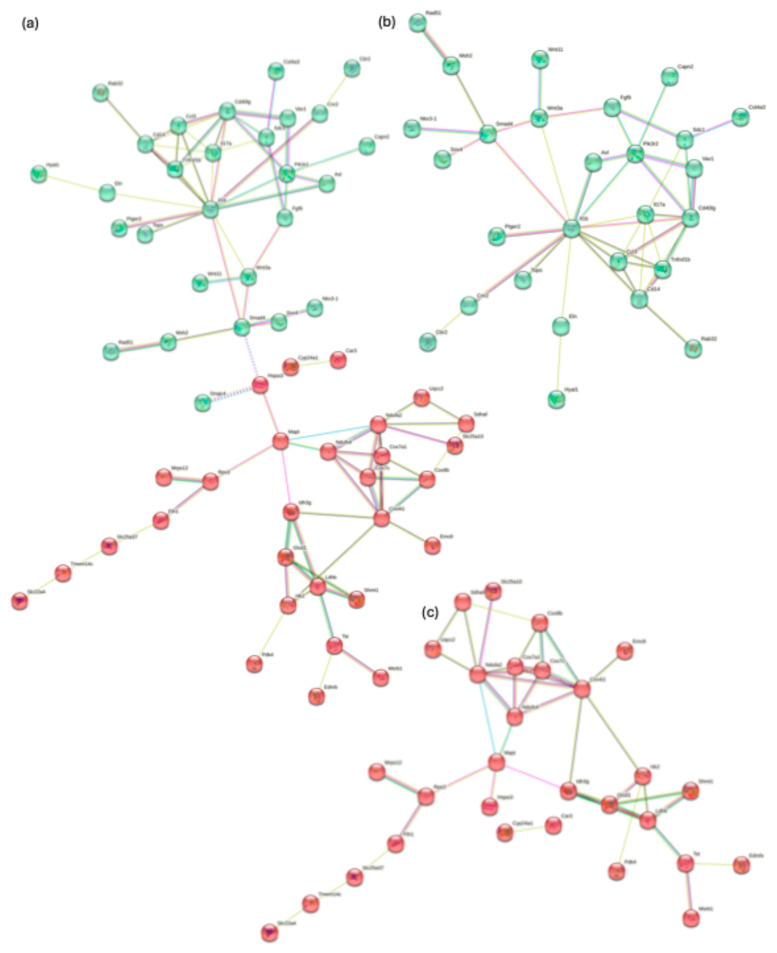
The analysis of the predicted protein–protein interaction. (**a**) STRING analysis shows the linkage of the neurodegenerative disease cluster with the cancer and immune response cluster. (**b**) Cluster 1 with enriched genes involved in metabolic pathways and neurodegenerative diseases. (**c**) Cluster 2 with enriched genes involved in cancer and immune response pathways.

For this reason, a cluster analysis was performed with the STRING database to create a protein–protein interaction network. The interaction network was mapped and visualized for upregulated and downregulated genes (Figure 6a). This figure shows the regulatory network altered in SAMP8 mice based on the interactions between 85 upregulated and downregulated mRNAs. Two clusters were mapped (Figure 6b,c) and visualized based on their interaction strength. Enrichment analyses of KEGG pathways of clusters were performed (Table 3 and Supplementary Table S3). Interestingly, metabolic pathways and neurodegenerative diseases were enriched for Cluster 1, while pathways in cancer and immune response processes were enriched for Cluster 2.

### 3.5. Change in the Expression of Genes Involved in Alzheimer’s Disease

**Figure 7:**
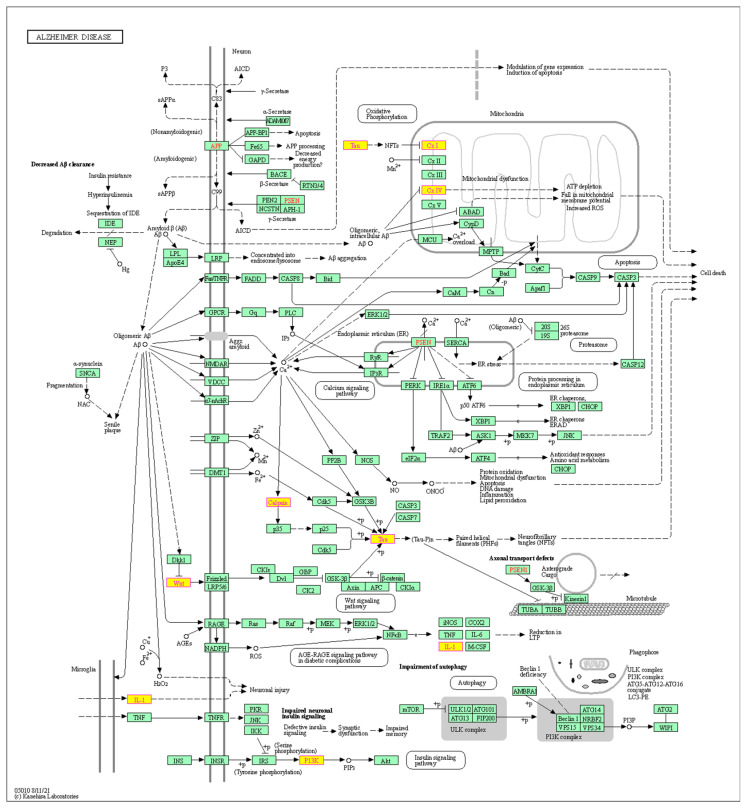
The expression profile of the SAMP8 mouse model shows changes in the expression levels of genes *Wnt*, *Calpin*, *IL-1*, *P13K*, *Tau*, *CxI*, *Cx IV*, and *Psen1*, which are involved in Alzheimer’s disease.

In the expression profile of the SAMP8 mice, changes were detected in the genes Wnt family (*Wnt*), Calpain (*Capn*), Interleukin 1 *(IL-1*), Phosphatidylinositol-3-kinase (P13K), Tau, Chemokine ligand (*CxI*), Chemokine IV (*Cx IV*), and Presenilin 1 (*Psen1*) which are involved in AD (Figure 7), demonstrating the relevance of the SAMP8 model as a model for sporadic AD.

### 3.6. Validation of the Microarray Expression Data

**Figure 8:**
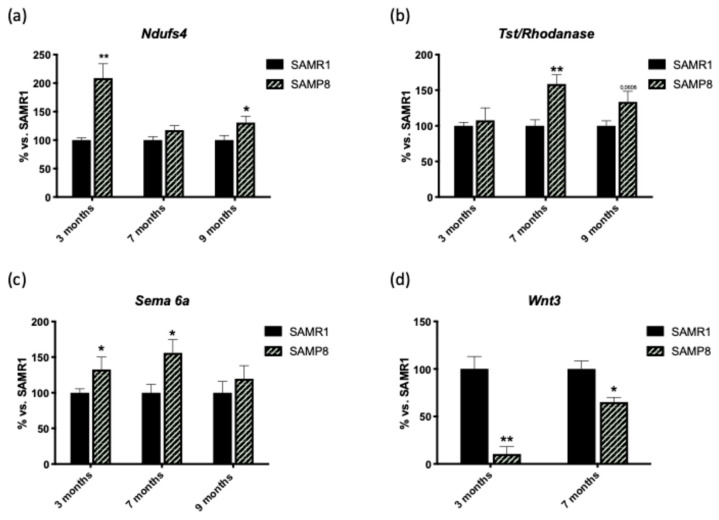
Validation of microarray expression data by RT-PCR for selected genes in SAMP8 and SAMR1 mice at different ages. (**a**) The expression of Ndufs4 was higher in SAMP8 mice than in SAMR1 mice at 3 and 9 months of age. (**b**) The expression of Tst/Rhodanase was significantly increased at 7 months of age and reached near significance at 9 months of age in the SAMP8 groups. (**c**) Sema6a expression was increased at 3 and 7 months of age in SAMP8 mice compared to SAMR1 mice. (**d**) Wnt3 gene expression was downregulated in SAMP8 mice in both age groups examined. Data are expressed as mean ± SEM. Statistical significance: * *p* < 0.05, ** *p* < 0.01 compared to age-matched SAMR1 mice. n = 4 per group.

To validate the results of the microarray analysis, mRNA candidates were selected from the upregulated and downregulated genes, and their relative expression was determined by qPCR. The selected genes were *NADH: ubiquinone oxidoreductase subunit S4* (*Ndufs4*), *TST/Rhodanese*, *Wnt* Family Member 3 *(Wnt3*), and *Semaphorin 6A* (*Sema6a*). The expression of *Ndufs4* was higher in SAMP8 mice at 3 and 9 months of age compared to SAMR1 mice (Figure 8a). The expression of *Tst/Rhodanase* increased significantly at 7 months of age and almost reached significance at 9 months of age in the SAMP8 groups (Figure 8b). The expression of *Sema6a* was increased at 3 and 7 months of age in SAMP8 mice compared to SAMR1 mice (Figure 8c). Finally, expression of the *Wnt3* gene was downregulated in SAMP8 at both ages examined (Figure 8d). These results are consistent with those of the microarray. As we can see, the expression of these genes goes in the same direction at all ages, but at certain ages, the change in expression is not significant. This shows that the expression of *Ndufs4*, *TST/Rhodanese*, *Wnt3*, and *Sema6a* were differentially expressed during aging in the SAMP8 mouse model.

## 4. Discussion

This study provides interesting findings about the accelerated aging process of SAMP8 mice compared to their healthy SAMR1 control mice. Bioinformatics analysis revealed that the SAMP8 strain has a number of genes whose expression is altered during aging. These genes can be broadly categorized into two main clusters: one group related to metabolic pathways and neurodegenerative diseases, and another group related to cancer pathways and immune responses. Analysis of the KEGG signaling pathway revealed that the differentially expressed genes in SAMP8 mice were mainly associated with three major categories: metabolic pathways and neurodegenerative diseases such as AD, were the most highly upregulated and downregulated differentially expressed genes, along with cancer pathways.

Metabolic pathways were the most altered category, indicating widespread changes in cellular metabolism in SAMP8 mice. Altered metabolic pathways likely contribute to the accelerated aging phenotype and increased susceptibility to neurodegenerative processes. Metabolic pathways affected include glucose metabolism, lipid metabolism, and energy production.

Among the neurodegenerative diseases, the pathways of AD were particularly strongly represented, which is consistent with the importance of SAMP8 model’s for AD research. In addition, other neurodegenerative diseases, such as Parkinson’s disease or Huntington’s disease, may also be affected suggesting common mechanisms in neurodegeneration. Genes in these signaling pathways likely contribute to the cognitive decline and neuropathological features observed in SAMP8 mice.

Finally, the involvement of cancer-related signaling pathways was an interesting and intriguing finding, suggesting possible links between aging, neurodegeneration, and cancer-associated processes. This could reflect dysregulation of cell cycle control, apoptosis, or DNA repair mechanisms that co-occur in cancer and neurodegenerative processes. We found that all of these signaling pathways were affected by both up- and downregulated genes, indicating complex regulatory changes in SAMP8 mice. For example, six upregulated genes (*Ndufa2*, *Ndufs4*, *Cox8b*, *Cox4i1*, *Cox7ai*, and *Cox7c*) were involved in both metabolic and AD-related pathways, highlighting the interconnectedness of these processes.

First, we examined the metabolic pathways, which are essential mediators of neuronal processes. Therefore, metabolic disturbances are considered as a key pathophysiological feature of early-stage neurodegenerative diseases, including AD [17]. Key metabolic disturbances include dysregulation of glucose metabolism, dysfunction of glycolysis, and pathogenesis of the pentose phosphate pathway, which play an essential role in the development of dementia [18]. One of the direct effects of these perturbations is ATP production, which ultimately leads to an insufficient energy supply to support normal neuronal processes [19]. Interestingly, six upregulated genes involved in this KEGG pathway (*Ndufa2*, *Ndufs4*, *Cox8b*, *Cox4i1*, *Cox7ai*, and *Cox7c*) were also involved in AD KEGG. On the other hand, these genes were involved in biological processes such as the electron transport chain, oxidative phosphorylation, and mitochondrial processes, further emphasizing the importance of regulating metabolic processes for neuronal health [20]. SAMP8 mice have been shown to have a hyperactive mitochondrial electron transport system from a young age that increases with age, leading to a higher generation of reactive oxygen species (ROS), which are critically involved in aging [21]. The hyperactive mitochondrial electron transport system could explain the over-expression of the gene *Ndufs4* observed in the hippocampus of SAMP8 mice compared to SAMR1 mice. This gene encodes for a subunit of complex I of the respiratory chain, a proton pump that mediates the transfer of electrons from NADH to ubiquinone (Q) [22,23]. Given its role in mitochondrial function and neurodegeneration, *Ndufs4* could be a potential target for therapeutic intervention in age-related cognitive decline and neurodegenerative diseases. Our results would therefore indicate which genes may be involved in altering mitochondrial function in SAMP8 mice, which may indeed partially explain the molecular but also phenotypic differences between SAMR1 and SAMP8 mice.

Another altered signaling pathway in SAMP8 was the immune system. The immune response influences the progression of neurodegeneration [24,25]. Redundant, persistent, and self-activating inflammatory processes in the brain are undoubtedly an important factor in promoting the progression of AD [25]. Our analysis revealed changes in genes associated with immune response pathways, which is consistent with the growing body of evidence linking neuroinflammation and neurodegenerative diseases. For instance, *Test/Rhodanese*, which encodes thiosulfate sulfurtransferase, plays an important role in sulfur metabolism and is involved in various physiological processes [22]. Although there is little evidence of a link between *Tst/Rhodanese* and neurodegeneration in the SAMP8 model, several studies suggest possible connections through its involvement in inflammation, oxidative stress, and mitochondrial function [26,27]. Interestingly, the SAMP8 mouse model shows increased neuroinflammation with increasing age [28]. Older SAMP8 mice (10 months old) show increased expression of pro-inflammatory markers such as *Il-1β*, *Tnf-α*, *Il-6*, and *Ccl2* in the brain [28]. In addition, SAMP8 mice exhibit alterations in signaling pathways related to oxidative stress and mitochondrial function [12], processes that involve *Tst/Rhodanese*. This partially suggests that the expression of *Tst/Rhodonase* in SAMP8 is increased during aging. This finding is supported by the work of Griñán-Ferré et al. (2016) [29], who demonstrated increased neuroinflammation in SAMP8 mice, characterized by high levels of pro-inflammatory cytokines and activated microglia. 

Changes in axon guidance molecules have been observed in various neurodegenerative diseases, including AD [30]. Semaphorins and their receptors are associated with different pathological conditions, including ischemia, degenerative diseases, and multiple sclerosis [31,32]. *Sema6a* was found over-expressed in the hippocampus of SAMP8 mice compared to SAMR1 mice. This gene encodes for a protein involved in several neuronal functions such as axonal guidance, neuron formation, and myelination of oligodendrocytes [22,33], its expression is higher in the postnatal brain but it is also over-expressed in demyelinating lesions [33]. Although *Sema6a* is not specifically mentioned, studies in SAMP8 mice have shown changes in the expression of genes related to axon guidance [34]. This suggests that axon guidance molecules such as semaphorins may play a role in age-related neurodegeneration. Accordingly, this type of lesion has been described as a process that accompanies aging and is exacerbated in AD [35,36], which reinforces the role of *Sema6a* in this feature. On the other hand, its over-expression has also been reported to favor the growth and survival of some cancers, such as BRAFV600E melanomas [37]. Under-expression of *Sema6a* has been reported in some other cancers, such as lung cancer and oral carcinoma. Therefore, the *Sema6a* gene regulates processes related to different types of cancer, such as cell migration [3,38].

Aging is associated with dysregulation of the Wnt signaling pathway, which affects ligands, downstream effectors, and Wnt target genes [39,40]. Similarly, dysregulation of Wnt signaling has been linked to various age-related diseases, including neurodegenerative diseases [40]. Remarkably, transcriptomic studies in SAMP8 mice have revealed changes in various signaling pathways with age, although Wnt signaling is not explicitly highlighted in the presented results [41]. Strikingly, we found reduced *Wnt3* gene expression in SAMP8 mice compared to SAMR1 mice as early as 3 months of age, which was maintained with increasing age. Following these results, protein levels of Wnt3a were significantly reduced in the hippocampus and cortex of aged *Octodon degus* (a rodent model of aging), compared to young animals [40]. Therefore, abnormal inhibition of Wnt signaling is associated with neurodegenerative diseases such as AD. Our findings suggest that the Wnt signaling pathway is involved in the neurodegenerative process in the SAMP8 model.

Consistent with our findings, other studies in SAMP8 mice coincide in identifying crucial pathways involved in aging and neurodegeneration, such as oxidative stress pathways (including oxidative stress and antioxidant response), apoptosis pathways, protein quality control (including pathways involved in protein folding and degradation), mitochondrial dysfunction (including mitochondrial function and energy production), cell metabolism (including genes related to glucose metabolism and cellular energy balance), neuroinflammation and inflammatory response (including expression of cytokines such as TNF-α, IL-6, and IL-1β), cellular senescence (including changes in cellular aging and senescence markers), and neurovascular interactions (affecting BBB integrity and vascular health on neurodegeneration) [41,42,43,44,45].

## 5. Conclusions

All these findings support the hypothesis that changes in gene expression of AD-related genes are responsible for the phenotypic differences between SAMR1 and SAMP8 mice during the aging process. In this context, it has been described how regulators of metabolism and the immune system, as well as genes associated with cancer pathways, may influence neuronal health and other processes that ultimately lead to these phenotypic alterations. These findings contribute to the growing body of evidence supporting the use of SAMP8 mice as a valuable model for studying age-related neurodegeneration. The altered gene expression profiles that we observed across different ages provide a molecular basis for the accelerated aging of these mice and the AD-like phenotypes. Thus, these processes are highly relevant to neurodegeneration and the SAMP8 model of accelerated aging. Future studies could validate these gene candidates and investigate their potential for therapeutic interventions for age-related cognitive decline and neurodegenerative diseases.

In summary, our study confirms that changes in the expression of AD-related genes, both by increasing or decreasing their expressions depending on specific genes, drive the phenotypic differences between SAMR1 and SAMP8 mice in aging. In particular, regulators of metabolism, immune response, and cancer-related genes influence neuronal health and contribute to these phenotypic changes. Our results emphasize the value of SAMP8 mice as a model for studying age-related neurodegeneration. The observed changes in gene expression at different ages provide a molecular basis for the accelerated aging and AD-like phenotypes in SAMP8 mice. These processes are crucial for understanding neurodegeneration. Future research should validate these gene candidates and explore their potential for developing therapeutic interventions against age-related cognitive decline and neurodegenerative diseases.

## Figures and Tables

**Table 1:** Genes downregulated and upregulated in the hippocampus of the SAMP8 mouse at 3, 7, and 9 months of age.

Downregulated Genes						Upregulated Genes					
3-Month-Old SAMP8		7-Month-Old SAMP8		9-Month-Old SAMP8		3-Month-Old SAMP8		7-Month-Old SAMP8		9-Month-Old SAMP8	
Gene	Z- Score	Gene	Z- Score	Gene	Z- Score	Gene	Z- Score	Gene	Z- Score	Gene	Z- Score
*Spire1*	−4.14	0610039K10Rik	−6.47	Gpr39	−5.36	Rnu5g	4.80	Rn18s	4.82	D16605	4.86
*Or8c9*	−4.09	Pclaf	−6.42	Inha	−4.72	Cyp24a1	4.73	Dnmt3a	4.73	Rny1	4.45
*5430421F17Rik*	−3.92	Mm2pr	−6.35	Neb	−4.71	Npff	4.53	X56974	4.25	Tnks2	4.27
*UGT2b37*	−3.89	M63850	−6.33	Psmd14	−4.55	A930007I19Rik	4.52	Stmn3	4.24	Sval1	4.21
*Agbl3*	−3.82	Slc6a21	−5.76	Agtr1a	−4.50	Rn18s	4.47	Kcnc1	4.13	Def6	4.19
*Dbr1*	−3.81	S100pbp	−5.58	Tbca	−4.45	Xlr	4.39	Add2	04.09	Rn18s	4.16
*1700001J11Rik*	−3.53	4930545L23Rik	−5.58	Sp6	−4.40	M19226	4.37	2610306M01Rik	3.96	Vpreb3	04.03
*Il22b*	−3.51	Gabrb1	−5.32	Trappc2b	−4.36	L37414	4.34	Anpep	3.82	Slc28a3	3.93
*IL22b*	−3.51	1700011C11Rik	−5.27	Invs	−4.34	Bbs9	4.32	5830462O15Rik	3.77	Krtap19-5	3.92
*AJ231271*	−3.48	S69343	−4.99	Med13I	−4.33	U24680	4.31	BE534640	3.75	Rny3	3.83

**Table 2:** Dysregulated genes in the hippocampus of the SAMP8 mouse at 3, 7, and 9 months of age included in the Venn diagram.

DISEASE	GENES
AGING	*Pik3ca*, *Irs4*, *Mtor*, *Tsc1*, *Sesn3*, *Pik3r2*, *Atf4*, *Rela*, *Ins1*, *Kl*, *Nfkb1*, *Eif4ebp1*, *Prkaca*
ALZHEIMER’S DISEASE	*Adam10*, *Cycs*, *Grin2b*, *Lrp1*, *Plcb3*, *Snca*, *Ndufs4*, *Ern1*, *Uqcrb*, *Cox7c*, *Cox8b*, *Il1b*, *Cox4i1*, *Cox5b*, *Cox7a1*, *Eif2ak3*, *Mapt*, *Ndufa1*, *Cox6a1*, *Ppp3cc*, *Capn2*, *Ndufa2*, *Atp2a3*, *Ndufb8*, *Atp5a1*, *Grin1*, *Mme*, *Plcb1*, *Ndufa12*, *Cacna1c*, *Cox6a2*, *Ndufb2*, *Casp12*, *Itpr1*, *Hsd17b10*
MITOCHONDRIA	*Gykl1*, *Rhot1*, *Tdh*, *Mrpl40*, *Slc25a14*, *Casp8ap2*, *Mrpl2*, *Tst*, *Pnkd*, *Mrpl10*, *Sox4*, *Hap1*, *Mrps12*, *Car5a*, *Rab11b*, *Pus1*, *Clpb*, *Slc25a20*, *Dnajc5*, *Prodh*, *Pex11b*, *Gnpat*, *Stap1*, *Suclg1*, *Cyp24a1*, *Dnajc4*, *Rad51*, *Gabarapl1*, *Pdk4*, *Fkbp8*, *Eln*, *1700123O20Rik*, *Ldhb*, *Smcp*, *Cryab*, *Slc25a26*, *Gcat*, *Emc8*, *Nipsnap1*, *Stoml2*, *Rai1*, *Endog*, *Ung*, *Uqcc2*, *Aldh2*, *Timm9*, *Slc25a30*, *Cbr2*, *Shmt1*, *Maff*, *Nme4*, *Sox4*
APOPTOSIS	*Pml*, *Casp6*, *Phlda1*, *Pglyrp1*, *Unc5c*, *Rabep1*, *Tgfbr1 Apip*, *Cycs*, *Zc3h8*, *Purb*, *Dfna5*, *Fkbp8 Mycs*, *Cul7 Prkd1*, *Rnf130*, *Sema6a*, *Dffb Inpp5d*, *Edar*, *Hipk3*, *Cdip1*, *Rassf7*, *Epb41l3*, *Csnk2a2*, *S100a14*, *Fem1b Melk*, *Aktip*, *Rybp*, *Sh3kbp1*, *Plscr1*, *Map2k7*, *Rtkn*, *Dcc*, *Tgfbr2*, *Polr2g*, *Ebag9*, *Fam188a*, *Rfk*, *Casp14*, *Vdac1*, *Ppid*, *Nfkb1*, *Emc4*, *Itgb3bp*, *Ercc2*, *Fam188a*, *Ctsc*, *Hyal*
INFLAMMATORY RESPONSE	*Hyal*, *Krt16*, *Kng*, *Tbxa Bdkr Chst*, *Rxra*, *Chil*, *Elf*, *Pik*, *Ciita*, *Csf*, *Prkd*, *Nos*, *Tlr Tnfr*, *Anxa*, *Il Ccl*, *Ucn Il Ccl Lxn*, *P Ptg*, *Klk*, *Cxcl Il*, *Npp*, *Cd Il*, *Ccl*, *Rela Il Ccl Il*, *Cd*, *Ccr*, *Itg*, *Agtr*, *Axl*, *Cnr*, *Cd*, *Cnr*, *Il Itg*, *Axl*, *Sdc*, *Cxcr*, *Nlr*, *Axl*, *Ly Cd*, *Nfkb*, *Myd*, *Bdkr*, *Ccl*, *Cnr*, *Ccl*, *Mif*, *Cx*, *Tg*, *Sdc*, *Ltb*, *Clec*, *Ccr*
OXIDATIVE STRESS	*Prnp*, *Msrb*, *Car*, *St*, *Ucn*, *Rps*, *Park*, *Jak*, *Gpx*, *Ndu*, *Chr*, *Psmb*, *Sel*, *Txnip*, *Ercc*
IMMUNE	*Endou*, *Mcpt*, *Vpre*, *Ct*, *Tin*, *Tlr*, *Zap*, *Tnf*, *Fth*, *Vpre*, *Ct Cc*, *l Cd H*, *Il Tinagl*, *Ccl*, *Tnfr Cd*, *Ltbr Il*, *Ccl*, *Vpre*, *Tnfsf11*, *Map*, *Mcpt*, *H Fth*, *Cxcl Il*, *Vpre*, *Cd Enpp*, *Il*, *Ccl*, *Ctsw*, *Il*, *Cd*, *Tnfsf8*, *H Azgp*, *Cd*, *Nfil*, *H Clnk*, *Nfil Prg*, *Myd Fcgr Vpre*, *Cxcl*, *Ccl*, *Bmpr*, *Vav*, *Ccr*, *Ccl*, *Cd274*, *Cx3cl Prg*, *H Q*, *W*
PATHWAYS OF CANCER	*Pml*, *Csf*, *Bdkr*, *Tgfbr*, *Cxcr*, *Wnt*, *Roc*, *Fzd*, *Mit Run*, *Gna*, *Mmp*, *Ret*, *Plcb*, *Cycs*, *Rxr*, *Mtor*, *Spi*, *Pik*, *Wnt*, *Rad*, *Smad*, *Bmp*, *Tgfb*, *Ral*, *Nkx*, *Gnb*, *Msh*, *Nos*, *Fzd*, *Fgf*, *Wnt*, *Map*, *Cycs*, *Fgf*, *Map*, *Pik*, *Gnb*, *Wnt*, *Fgf*, *Hhip*, *Veg*, *Rad*, *Jun*, *Pik*, *Msh*, *Ptge*, *Tcf*, *Nkx*, *Wnt*, *Gng*, *Smad*, *Plcb*, *Rela*, *Ptge*, *Itga*, *Birc*, *Run*, *Bmp*, *Hhip*, *Wnt*, *Mdm*, *Itga*, *Ptge*, *Dcc*, *Edn*, *Tgf*, *Col*, *Cs*, *Ct*, *Fzd*, *Gng*, *Rb*, *Col*, *Wnt*, *Max*, *Itga*, *Edn*, *Nfk*, *Hsp*, *Fgf*, *Bdkr*, *Tcf*, *Cxc*, *Ptk*, *Brca*, *Cd*, *Prk*, *Plcg*, *Wnt*, *Lamb*, *Fgf*, *Fgf*, *Tg*, *Gli*, *Fzd*, *Chu*, *Hsp*

**Table 3:** KEGG enrichment analyses of Cluster 1 and Cluster 2.

KEGG Enrichment Analyses of the Differentially Expressed Genes in the Clusters.						
Cluster	Description	Gene Count	Background Count	Strength	False Discovery Rate	Matching Proteins in Your Network (Labels)
1	Metabolic pathways	16	1536	0.75	2.07 × 10^−6^	*Hk2*, *Ndufa2*, *Shmt1*, *Ndufs4*, *Glud1*, *Cox8b*, *Car3*, *Ldhb*, *Cox4i1*, *Cyp24a1*, *Tst*, *Idh3g*, *Aadat*, *Cox7a1*, *Cox7c*, *Tusc3*
	Parkinson’s disease	7	239	1.2	3.52 × 10^−5^	*Ndufa2*, *Ndufs4*, *Cox8b*, *Cox4i1*, *Cox7a1*, *Mapt*, *Cox7c*
	Prion disease	7	264	1.15	4.05 × 10^−5^	*Hspa1l*, *Ndufa2*, *Ndufs4*, *Cox8b*, *Cox4i1*, *Cox7a1*, *Cox7c*
	Alzheimer’s disease	7	359	1.02	0.00022	*Ndufa2*, *Ndufs4*, *Cox8b*, *Cox4i1*, *Cox7a1*, *Mapt*, *Cox7c*
	Pathways in cancer	10	528	0.98	2.64 × 10^−5^	*Wnt3a*, *Fgf9*, *Nkx3-1*, *Msh2*, *Smad4*, *Rad51*, *Pik3r2*, *Ptger2*, *Col4a3*, *Wnt11*
	Huntington’s disease	6	296	1.04	0.00078	*Ndufa2*, *Ndufs4*, *Cox8b*, *Cox4i1*, *Cox7a1*, *Cox7c*
	Amyotrophic lateral sclerosis	6	364	0.95	0.0019	*Ndufa2*, *Ndufs4*, *Cox8b*, *Cox4i1*, *Cox7a1*, *Cox7c*
2	Pathways in cancer	10	528	0.98	2.64 × 10^−5^	*Wnt3a*, *Fgf9*, *Nkx3-1*, *Msh2*, *Smad4*, *Rad51*, *Pik3r2*, *Ptger2*, *Col4a3*, *Wnt11*
	Amoebiasis	5	105	1.38	0.00043	*Il1b*, *Pik3r2*, *Cd1d2*, *Cd14*, *Col4a3*
	Gastric cancer	5	147	1.23	0.0014	*Wnt3a*, *Fgf9*, *Smad4*, *Pik3r2*, *Wnt11*
	Toll-like receptor signaling pathway	4	98	1.31	0.0042	*Ccl3*, *Il1b*, *Pik3r2*, *Cd14*
	AGE-RAGE signaling pathway in diabetic complications	4	101	1.3	0.0042	*Smad4*, *Il1b*, *Pik3r2*, *Col4a3*
	Proteoglycans in cancer	5	199	1.1	0.0042	*Vav1*, *Wnt3a*, *Sdc1*, *Pik3r2*, *Wnt11*
	Cytokine–cytokine receptor interaction	5	279	0.95	0.0084	*Ccl3*, *Il17a*, *Il1b*, *Tnfrsf1b*, *Cd40lg*

## Data Availability

Data are contained within the article and Supplementary Materials.

## Supplementary Materials

The following supporting information can be downloaded at: https://www.mdpi.com/article/10.3390/genes15111411/s1, Table S1: Genes downregulated and upregulated in the hippocampus of the SAMP8 mouse at 3, 7, and 9 months; Table S2: Terms from the GO downregulated genes and analysis of upregulated; Table S3: KEGG enrichment analyses of Cluster 1 and Cluster 2.
